# An investigation on Alzheimer’s disease with obstructive sleep apnea: alterations of cognitive function, roles of cyclin-dependent kinase 5 and changes of brain structure

**DOI:** 10.3389/fnagi.2025.1552535

**Published:** 2025-12-01

**Authors:** Dongmei Luo, Tenghong Lian, Ning Wei, Peng Guo, Mingyue He, Yanan Zhang, Yue Huang, Gaifen Liu, Jinghui Li, Jing Li, Jing Qi, Huiying Guan, Wenjing Zhang, Weijia Zhang, Zijing Zheng, Hao Yue, Zhan Liu, Fan Zhang, Yao Meng, Wei Zhang

**Affiliations:** 1 Department of Neurology, Beijing Tiantan Hospital, Capital Medical University, Beijing, China; 2 Center for Cognitive Neurology, Department of Neurology, Beijing Tiantan Hospital, Capital Medical University, Beijing, China; 3 China National Clinical Research Center for Neurological Diseases, Beijing Tiantan Hospital, Capital Medical University, Beijing, China; 4 Department of Blood Transfusion, Beijing Tiantan Hospital, Capital Medical University, Beijing, China; 5 Department of Pharmacology, School of Medical Sciences Faculty of Medicine & Health, UNSW, Sydney, NSW, Australia; 6 Center of Parkinson’s Disease, Beijing Institute for Brain Disorders, Beijing, China; 7 Beijing Key Laboratory on Parkinson Disease, Beijing, China

**Keywords:** Alzheimer’s disease, obstructive sleep apnea, cyclin-dependent kinase 5, tau phosphorylation, synaptic dysfunction, brain structure

## Abstract

**Aims:**

To investigate alterations of cognitive function, roles of cyclin-dependent kinase 5 (CDK5) and changes of brain structure in Alzheimer’s disease (AD) with obstructive sleep apnea (OSA).

**Methods:**

Total 94 AD with OSA (AD-OSA) patients were divided into 49 cases of AD with mild OSA (AD-OSA-M) and 45 cases of AD with moderate and severe OSA (AD-OSA-MS). Demographic characteristics, cognitive function, the levels of AD neuropathological proteins, CDK5 and synaptic proteins in cerebrospinal fluid (CSF), and brain volume by magnetic resonance imaging were compared between the two groups. The correlations among OSA and the above variables were analyzed.

**Results:**

Compared with AD-OSA-M group, AD-OSA-MS group had a higher body mass index, lower scores of AVLT N7 and SCWT-C, longer SCWT-C time, higher levels of phosphorylated tau (P-tau) 396 and synaptophysin, lower CDK5 level and smaller volumes of brain gray and white matters in parts of frontal, parietal, temporal and occipital lobes. In AD-OSA patients, the decreased CDK5 level was correlated with the elevated levels of P-tau 396 and synaptophysin in CSF. In AD-OSA-MS group, reductions of gray matter and white matter volumes associated with OSA exacerbation was correlated with memory and executive function impairments. The *p*-values of above results were <0.05.

**Conclusion:**

In AD-OSA, OSA exacerbation is associated with memory impairment and executive dysfunction, P-tau 396 elevation and CDK5 decline in CSF, synaptic disruption, and brain atrophy. Additionally, CDK5 may represent a potential therapeutic target for the individuals with comorbid AD and OSA.

## 1 Introduction

Alzheimer’s disease (AD) is the most common type of cognitive impairment in the elderly, with the characteristics of progressive cognitive decline, neuropsychiatric symptoms, and impaired activities of daily living. Obstructive sleep apnea (OSA) is a common sleep disorder in the elderly, where partial or complete upper airway obstruction leads to sleep apnea during sleep, resulting in chronic intermittent hypoxia, frequent awakenings, sleep fragmentation, and ultimately, a significantly reduced sleep quality. Both AD and OSA significantly compromise life quality of the elderly. Multiple studies have shown that AD and OSA mutually increase the risk of each other (Chang et al., 2013; Jorge et al., 2019; Lutsey et al., 2018). Among AD patients, the prevalence of OSA reached up to 90.6%, which indicated that the population of co-morbid AD and OSA was huge and deserves our attention (Gaeta et al., 2020). However, there are currently no studies specifically targeting AD-OSA population. Existing cross-sectional studies have revealed no significant differences in cognitive function between AD with no OSA (AD-nOSA) group and AD with OSA (AD-OSA) group. However, as of now, there is no study regarding the influence of different degrees of OSA on the cognitive function in AD-OSA patients. Hence, this study aimed to compare and analyze the cognition, potential mechanisms, and brain structure of AD patients with mild OSA (AD-OSA-M) and moderate and severe OSA (AD-OSA-MS). Given that OSA is a manageable and treatable condition, further explorations of their relationship and underlying mechanisms may lead to the identification of new therapeutic targets for AD-OSA patients.

The main pathological hallmarks of AD include amyloid plaques and neurofibrillary tangles with β amyloid (Aβ) and phosphorylated tau (P-tau) as their corresponding major components and core biomarkers, leading to synaptic loss and neuronal degeneration. A previous study showed that the Aβ42 level in cerebrospinal fluid (CSF) from healthy controls was higher than those in OSA and AD patients, indicating the potential roles of OSA in the initiation and progression of AD pathology (Liguori et al., 2019). A longitudinal study indicated that OSA accelerated P-tau elevation in CSF from cognitively normal subjects and mild cognitive impairment (MCI) patients (Bubu et al., 2019). Thus, it is evident that OSA can promote the formation and progression of AD biomarkers of Aβ and P-tau, which underlying mechanisms, are however not fully elucidated, let alone the impact of different degrees of OSA on AD biomarkers.

Cyclin-dependent kinase 5 (CDK5) belongs to the family of serine/threonine kinases and is specifically expressed in nervous system. It plays crucial roles in neuronal growth, synaptic function, circadian rhythm, and neurodegeneration (Pao and Tsai, 2021). CDK5 is a critical kinase that promotes the pathological development and progression of AD. In the brains of postmortem AD patients, CDK5 level was found to be elevated (Lee et al., 1999), leading to an upregulation of the activities of presenilin 1 (PS1) and beta-site amyloid precursor protein cleaving enzyme 1 (BACE1), resulting in increased Aβ production and tau hyperphosphorylation (D’Mello, 2021). CDK5 inhibitors was showed to protect hippocampal neurons from AD pathology-induced damage, particularly against P-tau, thereby reducing neuronal degeneration and death (Maccioni et al., 2001). Hypoxia was identified as a key factor for CDK5 activation, and chronic intermittent hypoxia was a significant feature of OSA (Fang et al., 2019). However, there are no studies on the change of CDK5 level in the CSF from OSA patients, let alone from AD-OSA patients. Thus, we investigated CDK5 level in CSF and the roles it played on AD biomarkers in AD-OSA patients.

Synaptic impairment is an important pathophysiological change of AD. Synaptic proteins mainly include synaptophysin, synapsin I, and synaptosome associated protein 25 (SNAP-25). Synaptophysin, a membrane protein located on presynaptic vesicles, plays crucial roles in promoting the fusion of synaptic vesicles and presynaptic membranes, serving as a biomarker for synaptogenesis and remodeling. Synaptophysin level reflects synaptic density. It was observed that AD patients had decreased synaptophysin level in brains, indicating the impaired synaptic function in AD (Reddy et al., 2005). In a mouse model exposed to the condition mimicking OSA, chronic intermittent hypoxia compromised synaptic plasticity damage and accelerated neuronal apoptosis by down-regulating synaptophysin in hippocampus (Ying et al., 2023). Synapsin I is a synaptic protein that is responsible for regulating neurotransmitter release from presynaptic terminals and involved in maintaining synaptic plasticity (Longhena et al., 2021). Loss of synapsin I has been observed in the brains of AD patients (Qin et al., 2004). SNAP-25 is a synaptic protein that has multiple functions, including mediations of synaptic vesicle fusion, exocytosis and neurotransmitter release (Shin, 2014). It was found that SNAP-25 level in CSF from AD patients was dramatically increased, suggesting synaptic function was seriously impaired (Kivisäkk et al., 2022). Conversely, OSA patients exhibited decreased SNAP-25 level in CSF due to the possibly fluctuations of thoracic and intracranial pressures, hindering the flow of metabolite produced by neurons, including SNAP-25, from interstitial fluid to CSF (Ju et al., 2016). However, caution is warranted in interpreting this result as the study included only 10 cases of OSA patients (Ju et al., 2016). Despite this, the investigation of synaptic function in OSA and AD-OSA patients remains an unexplored field (Ju et al., 2016).

Brain structure can be evaluated by magnetic resonance imaging (MRI), with voxel-based morphometry (VBM) utilized to measure brain volume (Matsuda, 2016). The progression of AD was closely linked to the reduced gray matter volume (GMV), particularly in hippocampus, temporal, and frontal lobes (Wang et al., 2015). AD patients exhibited decreased white matter volume (WMV), particularly in bilateral hippocampus and inferior temporal gyrus, etc. (Yin et al., 2015). In OSA patients, two distinct patterns of brain structure changes were observed, in which, one pattern showed gray matter atrophy and white matter hyperintensity, and another pattern presented gray matter hypertrophy and restricted white matter diffusion, reflecting the response to chronic intermittent hypoxia, such as intracellular edema (Baril et al., 2021). Despite this, there is no study on the changes of brain structure in AD-OSA patients to date.

In this investigation, we recruited AD-OSA-M and AD-OSA-MS patients, assessed their cognitive function using various rating scales, measured the levels of AD biomarkers, CDK5, and synaptic proteins in CSF by enzyme-linked immunosorbent assay (ELISA), evaluated GMV and WMV by MRI, and conducted comparisons and correlation analyses among the aforementioned variables. The aims of this study were to understand the clinical characteristics, potential biomarkers and underlying mechanism of AD-OSA, and offer the future therapeutic targets for delaying the progression of AD.

## 2 Materials and methods

### 2.1 Ethics statement

This study was approved by the Review Board of Beijing Tiantan Hospital, Capital Medical University, and the written informed consents were obtained from all AD-OSA patients and their caregivers.

### 2.2 Subjects

#### 2.2.1 Inclusion criteria of AD and OSA

AD was diagnosed according to the 2011 National Institute of Aging and Alzheimer’s Disease criteria (NIA-AA) (McKhann et al., 2011).

Polysomnography (PSG) is the gold standard for the diagnosis of OSA. For AD patients capable of cooperation, PSG (Compumedics, Australia) was employed for OSA diagnosis (46.8%). For AD patients unable to tolerate PSG due to cognitive or behavioral symptoms, overnight actigraphy (Dehaier Medical Systems, China) and clinical symptoms assessments (nocturnal snoring and daytime sleepiness) were adopted for OSA diagnosis (53.2%). This dual-modal strategy maximized the feasibility and accuracy of diagnosis of OSA in AD patients. The overnight sleep diagnostic actigraphy has been widely used in clinical practice (Fekedulegn et al., 2020). Participants were requested to withdraw sleep medications for at least 12–14 h and sleep overnight in a quiet and comfortable environment (at the sleep center of our hospital). It analyzes sleep breathing through parameters of blood oxygen saturation, heart rate, snoring, and body movement, etc., and the diagnostic report covers variables including apnea–hypopnea index (AHI), awakenings, and types of breathing events, etc. We followed the clinical guidelines released by the American Academy of Sleep Medicine in 2017 (Qaseem et al., 2014; Kapur et al., 2017), and categorized AD-OSA patients into AD-OSA-M group with AHI of 5.0–14.9 events/hour and AD-OSA-MS group with AHI ≥15.0 events/hour based on AHI and the clinical symptoms (nocturnal snoring and daytime sleepiness).

#### 2.2.2 Exclusion criteria of AD and OSA

The exclusion criteria of AD and OSA were as follows: (1) Neurological diseases that might affect cognition besides AD, including cerebrovascular disease, Lewy bodies disease, Parkinson’s disease, frontotemporal degeneration, corticobasal degeneration, and epilepsy, etc. (2) The main types of sleep-disordered breathing were non-obstructive. (3) Articulation disorders, depression and mental illnesses that might affect emotional expression. (4) Unable to complete the rating scales assessments due to hearing and vision disorders and sleep monitoring due to severer cognitive or behavioral symptoms. (5) History of severe central nervous system diseases such as stroke.

#### 2.2.3 Collections of demographic and clinical information

Demographic information, including gender, age, disease duration and education level, and clinical information, including body mass index (BMI), smoking, drinking, apolipoprotein E (APOE) ε4 carrier, history of hypertension, hyperlipidemia, myocardial infarction, diabetes, mellitus, hyperlipidemia, cerebrovascular disease and thyroid disease, and family history of dementia were collected.

#### 2.2.4 Assessment of overall cognitive function

The overall cognitive function was assessed by using Mini-Mental State Examination (MMSE) (Folstein et al., 1975) and Montreal Cognitive Assessment (MoCA) (Nasreddine et al., 2005) scales.

MMSE scale is sensitive to dementia, which evaluates cognitive domains of orientation, memory, attention and numeracy, recall and language. The total score of MMSE scale is 30 points. The lower the score of MMSE scale, the worse the overall cognitive function. Patients with illiteracy, primary education, or more than junior education are identified as cognitive impairment when MMSE score is below 17, 20 or 24 points, respectively.

MoCA is sensitive to MCI, which assesses cognitive domains of attention and concentration, executive function, memory, language, visual-structural skills, abstract thinking, and calculation and orientation. The lower the score of MoCA scale, the worse the overall cognitive function. MoCA score ≤26 indicates cognitive impairment, and 1 point is added if the educational level of an individual is less than 12 years.

#### 2.2.5 Assessment of cognitive domain function

Verbal memory was evaluated by using Auditory Verbal Learning Test (AVLT) (Guo et al., 2009). During each trial, a list of 12 novel words is read aloud with a 1-second pause between each word, and subjects are asked to recall immediately as many words as possible. The above process is repeated 3 times, with scores named N1, N2, and N3, respectively. Subjects are then asked to recall the 12 words again after a 5-minute and 20-minute interval, with scores named N4 and N5, respectively. AVLT N1-3, AVLT N4 and AVLT N5 evaluate immediate recall, short-delayed recall and long-delayed recall, respectively. AVLT N6 tests logical memory and AVLT N7 rates ability of recognition. Visual delayed memory is evaluated by Rey–Osterreithm Complex Figure Test (RCFT)-delayed recall (Shin et al., 2006), during which, subjects are instructed to duplicate a complex figure within 10 minutes, with score named RCFT-imitation, and then draw the figure from memory after 30 minutes, with score named RCFT-delayed recall.

Language function was evaluated by using 30-item Boston Naming Test (BNT) (Katsumata et al., 2015). BNT involves displaying subjects with 30 pictures and asking subjects to name each one. One point is given for each picture correctly answered, and total number of correct answers is recorded. The lower the score of BNT, the severer the language dysfunction.

Visuospatial ability was evaluated by using RCFT-imitation. A low score of this test implies compromised visuospatial ability (Shin et al., 2006).

Attention was evaluated by using Stroop Color-Word Test-A (SCWT-A), SCWT-B (Bondi et al., 2002) and Trail Making Test-A (TMT-A) (Wei et al., 2018). In SCWT-A, there are 50 words, including red, yellow, blue and green, and subjects are instructed to read each word correctly and quickly. In SCWT-B, there are 50 circles with 4 colors of red, yellow, blue and green, and subjects are asked to read each color accurately and rapidly from left to right. TMT-A requires subjects to draw a line between 25 consecutive numbers as quickly as possible without lifting the pencil. The longer it takes to complete the above tests and the lower the scores of these tests, the worse the attention.

Executive function was evaluated by using SCWT-C (Bondi et al., 2002) and TMT-B (Wei et al., 2018). In SCWT-C, there are 50 words of different colors that do not correspond to color, and subjects are asked to read the colors of the words instead of the words themselves. In TMT-B, except for number 1, all the other 24 numbers appear twice, which are square and circle shapes, respectively. The longer they take to complete and the lower the scores of these tests, the worse the executive function.

#### 2.2.6 Collections of CSF samples

CSF samples were obtained by lumbar puncture by using standard sterile techniques between 7–10 a.m. A total of 5 mL CSF was collected in a polypropylene tube (Beijing Jingke Hongda Biotechnology Co., China). CSF samples were immediately centrifuged at 3,000 r/min for 10 min after collection at 4 °C. Each CSF sample was then aliquoted into separate Nunc cryotubes (Beijing Jingke Hongda Biotechnology Co., China) and frozen for 1.0 mL per tube at −80 °C until the assay.

#### 2.2.7 Measurements of AD biomarkers, CDK5 and synaptic proteins in CSF

The levels of AD biomarkers, including Aβ42 (CUSABIO Company, China), P-tau 181 (Invitrogen, United States), P-tau 199 (Invitrogen, United States), P-tau 231 (Invitrogen, United States) and P-tau 396 (Invitrogen, United States), in CSF were measured by ELISA.

The level of CDK5 (NOVUS, United States) in CSF was measured by ELISA.

The levels of synaptic proteins, including synaptophysin (NOVUS, United States), synapsin-I (Signalway Antibody LLC, United States) and SNAP-25 (Abcam, United States), in CSF were measured by ELISA.

#### 2.2.8 Evaluations of GMV and WMV by MRI

All patients completed cranial MRI examination within approximately 1 week before and after the lumbar puncture. Patients were scanned on a 3.0 T MRI scanner (Siemens, Germany), and 3-dimensional magnetization prepared rapid-acquisition gradient echo sequence was acquired with the following parameters: repetition time = 2,300 ms, echo time = 2.3 ms, inversion time = 900 ms, flip angle = 8°, slice thickness = 1 mm, voxel size = 1 × 1 × 1 mm³, matrix = 256 × 256, FOV = 256 mm, parallel imaging (GRAPPA) factor = 2, and bandwidth = 200 Hz/pixel.

VBM was used to collect brain structure information. Firstly, dcm2niix was used to convert all image data format from DICOM to NIFTI format. Then, CAT12 were used to preprocess the structural data. The whole brain was divided into 170 regions of interest (ROI) based on the automated anatomical labeling atlas 3 (Rolls et al., 2020), and then GMVs and WMVs of ROIs were extracted.

### 2.3 Statistical analysis

Data were analyzed using SPSS Statistics 26.0 (IBM Corporation, United States), R version 4.3.2 (RStudio, United States). Graphical representation of the data was performed using the GraphPad Prism 9 (GraphPad Software, United States) software and the Xiantao online tool.^1^ Statistical significance was defined as a two-sided *p* < 0.05. Data were tested for normal distribution by using the Kolmogorov–Smirnov test. Demographic and clinical information, cognitive function, the levels of AD biomarkers, CDK5, and synaptic proteins in CSF, GMV and WMV between AD-OSA-M and AD-OSA-MS groups were compared. Continuous variables conforming to normal distribution were presented as means ± standard deviations (SD) and compared by two-tailed *t*-test, while non-normal distributed variables were presented as median (quartile) and compared by nonparametric test. Categorical variables were presented as number (percentage) and compared by chi-squared test. Multiple linear regression was used to assess the association after adjusting covariates. Spearman or Pearson correlation analysis was used to evaluate the correlations among the variables measured.

## 3 Results

### 3.1 The frequencies of different degrees of OSA in AD-OSA patients

**Figure 1:**
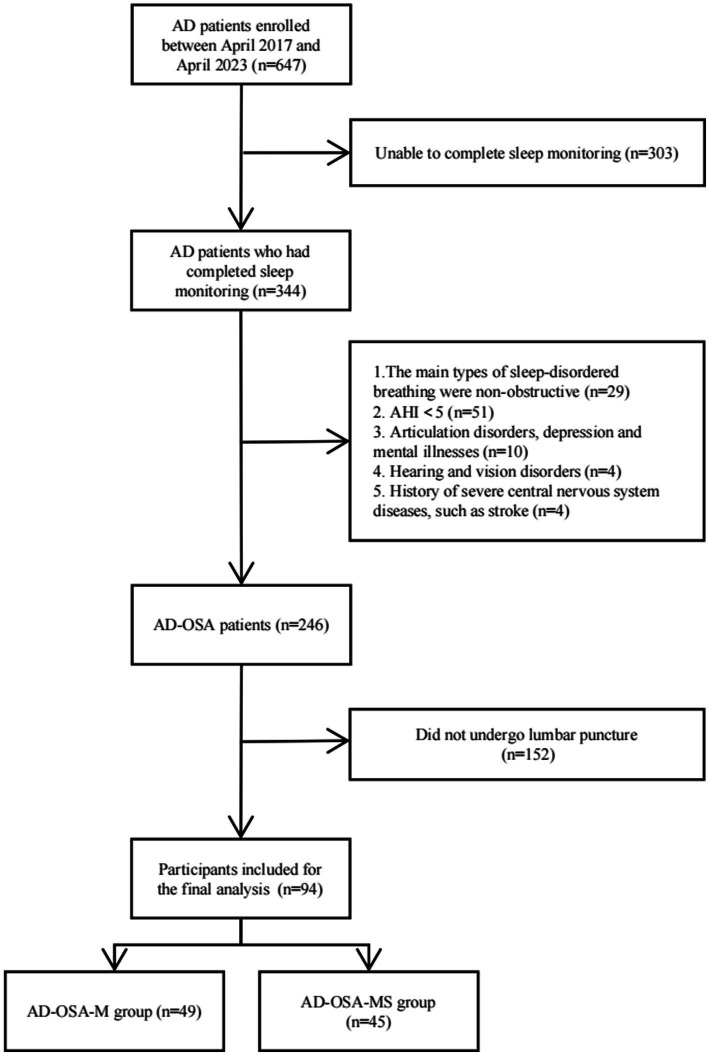
Study flow chart. This chart depicted the detailed recruitment and analysis processes. This study continuously included 647 AD patients from April 2017 to April 2023. Patients who were unable to complete sleep monitoring, did not meet the inclusion criteria, or did not undergo lumbar puncture were excluded. A total of 94 patients were included in the final analysis. AD, Alzheimer’s disease; AHI, apnea–hypopnea index; AD-OSA, Alzheimer’s disease with obstructive sleep apnea; AD-OSA-M, Alzheimer’s disease with mild obstructive sleep apnea; AD-OSA-MS, Alzheimer’s disease with moderate and severe obstructive sleep apnea.

This study continuously included 647 patients diagnosed with AD from April 2017 to April 2023. In accordance with the established inclusion and exclusion criteria. Eventually, 94 patients were included in the final analysis (Figure 1). In 94 AD-OSA patients, 49 cases had mild OSA with a frequency of 52.13%; 32 cases had moderate OSA with a frequency of 34.04%, and 13 cases had severe OSA with a frequency of 13.83%, resulting in 45 cases having moderate and severe OSA with a frequency of 47.87% (Table 1).

**Table 1:** Comparisons of demographic and clinical information between AD-OSA-M and AD-OSA-MS groups.

Demographic and clinical information	AD-OSA-M group (*n* = 49)	AD-OSA-MS group (*n* = 45)	*p*
Demographic information			
Female [*n* (%)]	26 (53.06)	22 (48.89)	0.686
Age (years, mean ± SD)	64.27 ± 9.06	67.32 ± 9.23	0.050
Disease duration [months, median (quartile)]	24.00 (12.00, 48.00)	24.50 (13.50, 48.00)	0.882
Education level			0.539
Primary school and below [*n* (%)]	12 (18.44)	11 (24.44)	
Middle and high school [*n* (%)]	27 (55.10)	24 (53.33)	
Bachelor’s degree and above [*n* (%)]	10 (20.41)	10 (22.22)	
Clinical information			
BMI (kg/m^2^, mean ± SD)	22.53 ± 2.78	24.88 ± 2.77	0.000^**^
Smoking [*n* (%)]	14 (28.57)	13 (28.89)	0.628
Drinking [*n* (%)]	15 (30.61)	11 (24.44)	0.481
APOE ε4 carriers [*n* (%)]	6 (12.50)	6 (13.33)	0.905
History			
Hypertension [*n* (%)]	14 (29.17)	16 (35.56)	0.510
Hyperlipidemia [*n* (%)]	12 (25.00)	6 (13.33)	0.155
Myocardial infarction [*n* (%)]	0 (0.00)	1 (2.22)	0.304
Diabetes mellitus [*n* (%)]	7 (14.58)	6 (13.33)	0.862
Hyperhomocysteinemia [*n* (%)]	1 (2.08)	2 (4.44)	0.520
Cerebrovascular disease [*n* (%)]	6 (12.77)	7 (15.56)	0.701
Thyroid disease [*n* (%)]	3 (6.25)	2 (4.44)	0.700
Family history of dementia [*n* (%)]	17 (37.78)	11 (27.50)	0.314

Data were presented as number (percentage), means ± SD or median (quartile). ^**^p < 0.01. AD-OSA-M, Alzheimer’s disease with mild obstructive sleep apnea; AD-OSA-MS, Alzheimer’s disease with moderate and severe obstructive sleep apnea; SD, standard deviation; BMI, body mass index; APOE, apolipoprotein E.

### 3.2 Demographic information of AD-OSA-M and AD-OSA-MS groups

Demographic information was compared between AD-OSA-M and AD-OSA-MS groups. It was indicated that BMI in AD-OSA-MS group was significantly higher than that in AD-OSA-M group (*p* < 0.05). The remaining demographic variables, such as age, gender, disease duration, education level, smoking, and drinking, did not show significant differences between the two groups (Table 1).

### 3.3 Cognitive function of AD-OSA-M and AD-OSA-MS groups

Comparisons of cognitive function between AD-OSA-M and AD-OSA-MS groups were performed. It was found that the scores of AVLT N7 and SCWT-C were significantly lower, and the SCWT-C time was significantly longer in AD-OSA-MS group than those in AD-OSA-M group (all *p* < 0.05). There were no significant differences in the scores of MMSE, MoCA, AVLT N1-6, BNT, RCFT, SCWT-A, SWCT-B, and TMT scales between the two groups (Table 2).

**Table 2:** Comparisons of cognitive function between AD-OSA-M and AD-OSA-MS groups.

Cognitive function	AD-OSA-M group (*n* = 49)	AD-OSA-MS group (*n* = 45)	*p*
Overall cognitive function			
MMSE [points, median (quartile)]	19.00 (11.50, 24.00)	19.00 (10.00, 24.00)	0.491
MoCA (points, mean ± SD)	13.13 ± 5.85	11.93 ± 5.93	0.337
Individual cognitive domain function			
Memory			
AVLT N1-3 (points, mean ± SD)	10.27 ± 6.23	8.80 ± 4.94	0.229
AVLT N4 [points, median (quartile)]	0.00 (0.00, 3.00)	0.00 (0.00, 2.00)	0.885
AVLT N5 [points, median (quartile)]	0.00 (0.00, 2.00)	0.00 (0.00, 2.00)	0.775
AVLT N6 [points, median (quartile)]	0.00 (0.00, 2.00)	0.00 (0.00, 1.00)	0.388
AVLT N7 [points, median (quartile)]	10.00 (6.00, 12.00)	6.50 (0.00, 9.00)	0.007^**^
RCFT-delayed [points, median (quartile)]	0.00 (0.00, 6.00)	0.00 (0.00, 6.00)	0.548
Language			
BNT [points, median (quartile)]	21.00 (18.00, 26.00)	20.00 (17.75, 25.00)	0.471
Visuospatial ability			
RCFT-imitation [points, median (quartile)]	25.00 (2.00, 32.75)	12.25 (0.00, 30.00)	0.124
Attention			
SCWT-A [points, median (quartile)]	50.00 (49.00, 50.00)	50.00 (46.00, 50.00)	0.352
SCWT-A (time) [seconds, median (quartile)]	47.00 (34.00, 80.00)	46.00 (36.50, 78.50)	0.992
SCWT-B [points, median (quartile)]	50.00 (48.00, 50.00)	49.00 (45.00, 50.00)	0.267
SCWT-B (time) [seconds, median (quartile)]	61.00 (46.00, 100.00)	66.00 (46.00, 120.25)	0.426
TMT-A [points, median (quartile)]	25.00 (20.50, 25.00)	24.00 (18.50, 25.00)	0.214
TMT-A (time) [seconds, median (quartile)]	116.83 (75.25, 239.25)	146.00 (71.50, 240.00)	0.295
Executive function			
SCWT-C [points, median (quartile)]	47.00 (30.00, 49.00)	41.00 (35.00, 46.50)	0.037^*^
SCWT-C (time) [seconds, median (quartile)]	108.00 (90.00, 153.00)	145.00 (98.00, 240.00)	0.039^*^
TMT-B [points, median (quartile)]	20.00 (0.00, 25.00)	12.00 (1.50, 22.50)	0.270
TMT-B (time) [seconds, median (quartile)]	240.00 (165.50, 240.00)	240.00 (210.00, 240.00)	0.139

Data were presented as mean ± SD or median (quartile). ^*^p < 0.05 and ^**^p < 0.01. AD-OSA-M, Alzheimer’s disease with mild obstructive sleep apnea; AD-OSA-MS, Alzheimer’s disease with moderate and severe obstructive sleep apnea; SD, standard deviation; MMSE, Mini-Mental State Examination; MoCA, Montreal Cognitive Assessment; AVLT, Auditory Verbal Learning Test; RCFT, Rey–Osterrieth Complex Figure Test; BNT, Boston Naming Test; SCWT, Stroop Color and Word Test; TMT, Trail Making Test.

**Figure 2:**
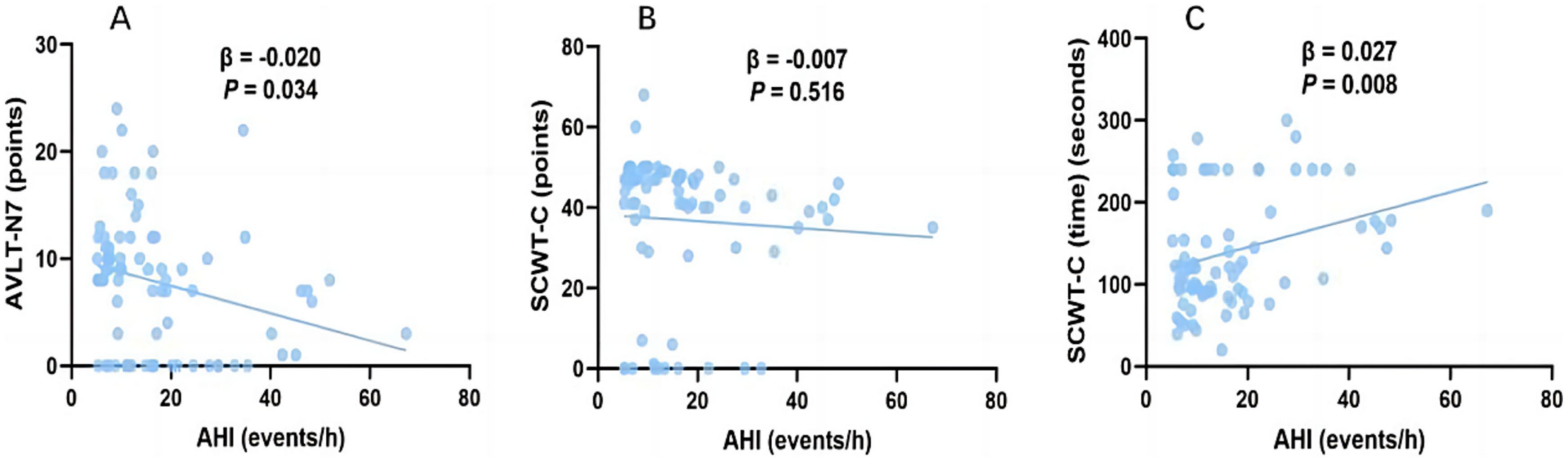
The association between AHI and cognitive function in AD-OSA patients. Multiple linear regression analysis between AHI and AVLT-N7 **(A)**, SCWT-C **(B)**, and SCWT-C (time) **(C)** in AD-OSA patients. Age, gender, and BMI were adjusted. AD-OSA, Alzheimer’s disease with obstructive sleep apnea; AHI, apnea–hypopnea index; events/h, events/hour; AVLT, Auditory Verbal Learning Test; SCWT, Stroop Color and Word Test; β, beta; CI, confidence interval.

After adjusting for confounding factors, (including age, sex and BMI), by multiple linear regression, higher AHI was significantly correlated with lower AVLT N7 score [*β*, −0.020; 95% CI (−0.039, −0.002); *p* = 0.034] and longer SCWT-C time [*β*, 0.027; 95% CI (0.007, 0.047); *p* = 0.008] in AD-OSA patients (Figure 2 and Supplementary Table 1).

### 3.4 AD biomarkers levels in CSF of AD-OSA-M and AD-OSA-MS groups

All patients in AD-OSA-M group and AD-OSA-MS group underwent lumbar puncture. The levels of AD biomarkers, including Aβ42, P-tau 181, P-tau 199, P-tau 231, P-tau 396, and T-tau in CSF were compared between AD-OSA-M and AD-OSA-MS groups. It was observed that AD-OSA-MS group had significantly elevated P-tau 396 level in CSF compared to AD-OSA-M group (*p* < 0.05). There were no significant differences in the levels of Aβ42, P-tau 181, P-tau 199, P-tau 231, and T-tau in CSF between two groups (Table 3).

**Table 3:** Comparisons of the levels of AD biomarkers, CDK5, and synaptic proteins in CSF between AD-OSA-M and AD-OSA-MS groups.

AD biomarkers, CDK5 and synaptic proteins	AD-OSA-M group (*n* = 49)	AD-OSA-MS group (*n* = 45)	*p*
AD biomarkers			
Aβ42 [ng/mL, median (quartile)]	0.52 (0.33, 1.06)	0.84 (0.35, 1.56)	0.282
P-tau 181 [pg/mL, median (quartile)]	38.14 (27.85, 85.81)	55.36 (33.10, 86.54)	0.202
P-tau 199 [pg/mL, median (quartile)]	6.30 ± 3.25	7.03 ± 3.18	0.349
P-tau 231 [pg/mL, median (quartile)]	88.19 ± 32.05	87.77 ± 33.70	0.959
P-tau 396 (pg/mL, mean ± SD)	56.32 ± 23.41	68.68 ± 25.82	0.038^*^
CDK5 protein			
CDK5 [ng/mL, median (quartile)]	1.06 (0.55, 1.33)	0.22 (0.12, 1.07)	0.006^**^
Synaptic proteins			
Synaptophysin [pg/mL, median (quartile)]	157.18 (128.18, 190.40)	250.97 (176.18, 405.55)	0.001^**^
Synapsin I [ng/mL, median (quartile)]	0.13 ± 0.05	0.13 ± 0.06	0.838
SNAP-25 (pg/mL, mean ± SD)	66.80 ± 23.59	78.58 ± 36.01	0.863

Data were presented as means ± SD or median (quartile). ^*^p < 0.05 and ^**^p < 0.01. AD-OSA-M, Alzheimer’s disease with mild obstructive sleep apnea; AD-OSA-MS, Alzheimer’s disease with moderate to severe obstructive sleep apnea; SD, standard deviation; Aβ, β amyloid; P-tau, phosphorylated tau; CDK5, cyclin-dependent kinase 5; SNAP-25, synaptosomal-associated protein 25.

**Figure 3:**
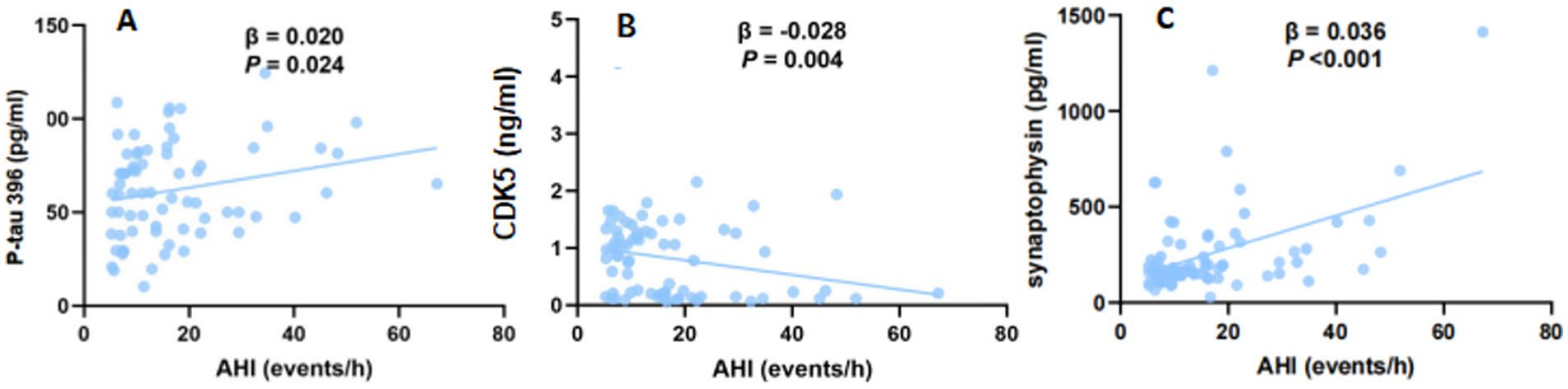
The association between AHI and the levels of P-tau 396, CDK5, and synaptophysin in the CSF from AD-OSA patients. Multiple linear regression analysis between AHI and the levels of P-tau 396 **(A)**, CDK5 **(B)**, and synaptophysin **(C)** in the CSF from AD-OSA patients. Age, gender, and BMI were adjusted. AD-OSA, Alzheimer’s disease with obstructive sleep apnea; AHI, apnea–hypopnea index; events/h, events/hour; CSF, cerebrospinal fluid; P-tau, phosphorylated tau; CDK5, cyclin-dependent kinase 5; β, beta; CI, confidence interval.

After adjusting for confounding factors, including age, sex and BMI, by multiple linear regression, the increased AHI was significantly correlated with the elevated P-tau 396 level in the CSF from AD-OSA patients [*β*, 0.020; 95% CI (0.003, 0.038); *p* = 0.024] (Figure 3 and Supplementary Table 2).

### 3.5 CDK5 level in CSF of AD-OSA-M and AD-OSA-MS groups, and the correlation between CDK5 and P-tau 396 levels in AD-OSA patients

CDK5 level in CSF was compared between AD-OSA-M and AD-OSA-MS groups. It was presented that CDK5 level in CSF was significantly decreased in AD-OSA-MS group compared with AD-OSA-M group (*p* < 0.05) (Table 3).

After adjusting for confounding factors, including age, sex, and BMI, by multiple linear regression, the increased AHI was significantly correlated with the decreased CDK5 level in the CSF from AD-OSA patients [*β*, −0.028; 95% CI (−0.047, −0.009); *p* = 0.004] (Figure 3 and Supplementary Table 2).

**Figure 4:**
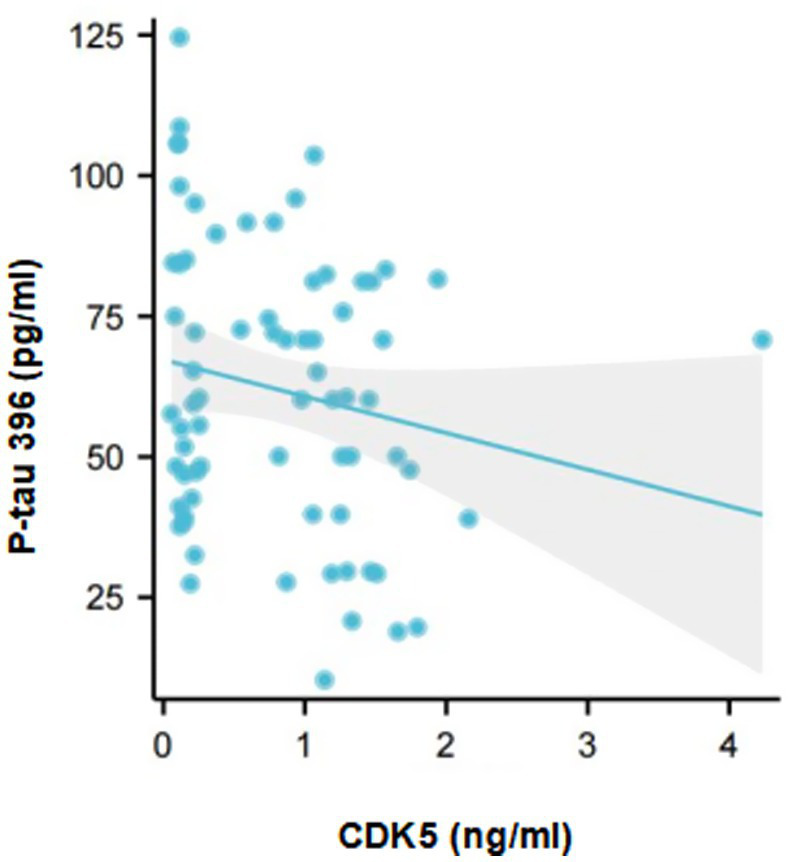
The correlation between CDK5 and P-tau 396 levels in the CSF from AD-OSA patients. AD-OSA, Alzheimer’s disease with obstructive sleep apnea; CDK5, cyclin-dependent kinase 5; P-tau, phosphorylated tau.

Furthermore, the correlation between CDK5 and P-tau 396 level in CSF from AD-OSA patients was analyzed. It was found that CDK5 level was significantly and negatively correlated with P-tau 396 levels in the CSF from AD-OSA patients (*r* = −0.246, *p* = 0.039) (Figure 4).

### 3.6 Levels of synaptic proteins in CSF of AD-OSA-M and AD-OSA-MS groups, and the correlation between CDK5 and synaptophysin levels in AD-OSA patients

The levels of synaptic proteins, including synaptophysin, synapsin I, and SNAP-25 in CSF were compared between AD-OSA-M and AD-OSA-MS groups. AD-OSA-MS group exhibited a significantly increased synaptophysin level in CSF compared to AD-OSA-M group (*p* < 0.05). There were no significant differences in synapsin I and SNAP-25 levels in CSF between the two groups (Table 3).

After adjusting for confounding factors (including age, sex and BMI) by multiple linear regression, increased AHI was significantly correlated with higher synaptophysin level in CSF from AD-OSA patients [*β*, 0.036; 95% CI (0.018, 0.053); *p* < 0.001] (Figure 3).

**Figure 5:**
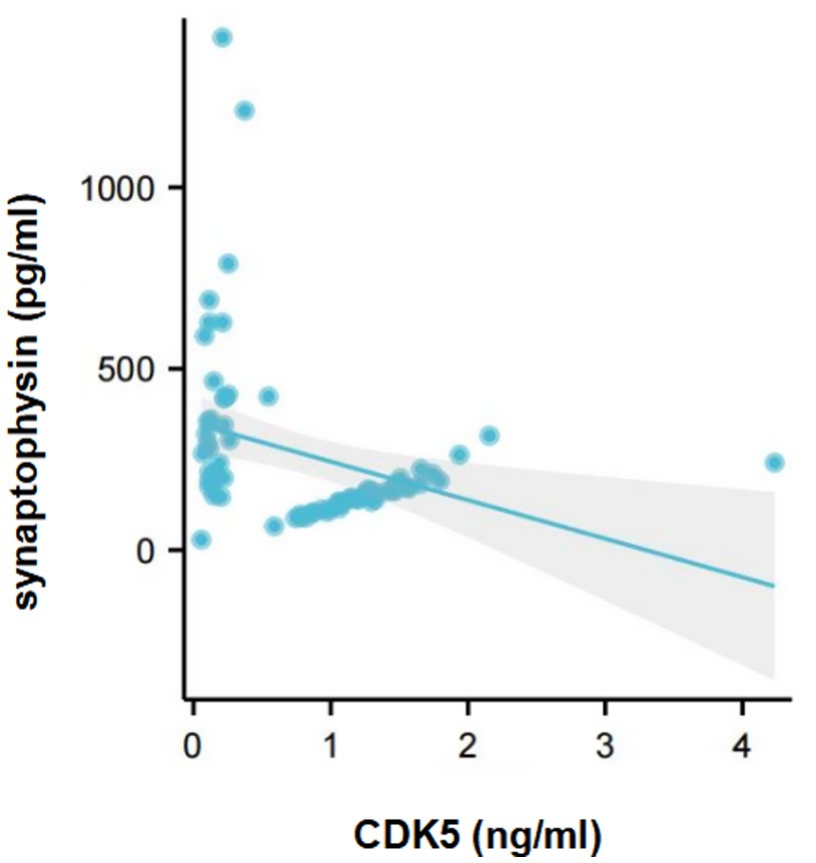
The correlation between CDK5 and synaptophysin levels in the CSF from AD-OSA patients. AD-OSA, Alzheimer’s disease with obstructive sleep apnea; CDK5, cyclin-dependent kinase 5.

Furthermore, the correlation between CDK5 and synaptophysin levels in CSF from AD-OSA patients was performed. It was found that CDK5 level was significantly and negatively correlated with synaptophysin level in CSF from AD-OSA patients (*r* = −0.327, *p* = 0.005) (Figure 5).

### 3.7 Brain volume of AD-OSA-M and AD-OSA-MS groups, and the correlations among AHI, cognitive function, and brain volume

#### 3.7.1 GMV

The GMV of 170 brain regions were compared between AD-OSA-M and AD-OSA-MS groups. AD-OSA-MS group showed significantly reduced GMV in 17 regions compared with AD-OSA-M group, including the left triangular part of inferior frontal gyrus, right rolandic operculum, right posterior orbitofrontal cortex, bilateral lingual gyrus, left superior occipital gyrus, right fusiform gyrus, right postcentral gyrus, left supramarginal gyrus, left superior temporal gyrus, bilateral temporal pole of superior temporal gyrus, left middle temporal gyrus, left temporal pole of middle temporal gyrus, bilateral inferior temporal gyrus, right medial geniculate nucleus (all *p* < 0.05). The GMVs of other brain regions did not differ significantly between two groups (Table 4).

**Table 4:** Comparisons of the GMVs of ROIs between AD-OSA-M and AD-OSA-MS groups.

GMV	AD-OSA-M group (*n* = 42)	AD-OSA-MS group (*n* = 30)	*p*
Left triangular part of inferior frontal gyrus (mm^3^, mean ± SD)	6.43 ± 0.90	5.99 ± 0.85	0.045^*^
Right rolandic operculum (mm^3^, mean ± SD)	3.82 ± 0.56	3.56 ± 0.50	0.043^*^
Right posterior orbitofrontal cortex (mm^3^, mean ± SD)	1.49 ± 0.22	1.38 ± 0.20	0.045^*^
Left lingual gyrus (mm^3^, mean ± SD)	6.33 ± 0.89	5.88 ± 0.80	0.033^*^
Right lingual gyrus (mm^3^, mean ± SD)	6.59 ± 0.95	6.16 ± 0.78	0.047^*^
Left superior occipital gyrus (mm^3^, mean ± SD)	2.96 ± 0.49	2.71 ± 0.48	0.039^*^
Right fusiform gyrus (mm^3^, mean ± SD)	9.25 ± 1.39	8.51 ± 1.06	0.013^*^
Right postcentral gyrus (mm^3^, mean ± SD)	9.02 ± 1.26	8.40 ± 0.96	0.027^*^
Left supramarginal gyrus (mm^3^, mean ± SD)	3.94 ± 0.57	3.62 ± 0.46	0.014^*^
Left superior temporal gyrus (mm^3^, mean ± SD)	6.27 ± 0.87	5.76 ± 0.84	0.016^*^
Left temporal pole of superior temporal gyrus (mm^3^, mean ± SD)	3.64 ± 0.58	3.30 ± 0.62	0.024^*^
Right temporal pole of superior temporal gyrus (mm^3^, mean ± SD)	3.83 ± 0.56	3.49 ± 0.52	0.011^*^
Left middle temporal gyrus (mm^3^, mean ± SD)	14.59 ± 2.35	13.29 ± 2.60	0.031^*^
Left temporal pole of middle temporal gyrus (mm^3^, mean ± SD)	2.55 ± 0.49	2.27 ± 0.47	0.021^*^
Left inferior temporal gyrus (mm^3^, mean ± SD)	10.52 ± 1.87	9.50 ± 1.83	0.025^*^
Right inferior temporal gyrus (mm^3^, mean ± SD)	10.82 ± 1.67	9.95 ± 1.46	0.025^*^
Right medial geniculate nucleus (mm^3^, mean ± SD)	0.029 ± 0.006	0.026 ± 0.006	0.036^*^

Data were presented as means ± SD. ^*^p < 0.05. GMV, gray matter volume; AD-OSA-M, Alzheimer’s disease with mild obstructive sleep apnea; AD-OSA-MS, Alzheimer’s disease with moderate and severe obstructive sleep apnea; SD, standard deviation.

**Figure 6:**
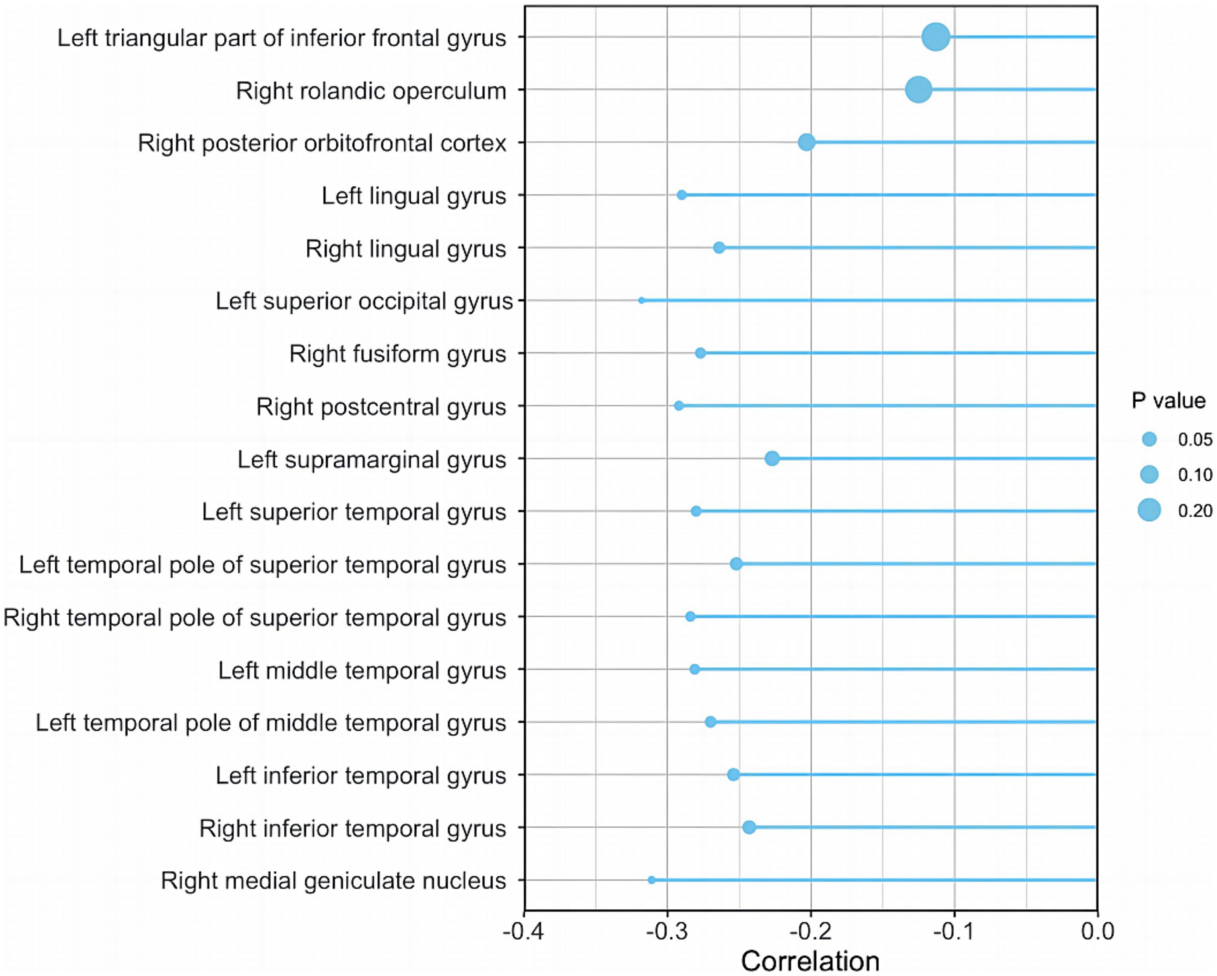
The correlation between AHI and the GMVs of ROIs in AD-OSA patients. AD-OSA, Alzheimer’s disease with obstructive sleep apnea; AHI, apnea–hypopnea index; GMV, gray matter volume.

It is well known that the higher the AHI, the more respiratory events occur during sleep, and the more severe the OSA. We further compared the correlation between AHI and the GMVs of the above 17 brain regions of AD-OSA patients. It revealed that AHI level was significantly and negatively correlated with the GMVs of left lingual gyrus (*r* = −0.290, *p* = 0.014), right lingual gyrus (*r* = −0.264, *p* = 0.026), left superior occipital gyrus (*r* = −0.318, *p* = 0.007), right fusiform gyrus (*r* = −0.277, *p* = 0.019), right postcentral gyrus (*r* = −0.292, *p* = 0.013), left superior temporal gyrus (*r* = −0.280, *p* = 0.018), left temporal pole of superior temporal gyrus (*r* = −0.252, *p* = 0.034), right temporal pole of superior temporal gyrus (*r* = −0.284, *p* = 0.016), left middle temporal gyrus (*r* = −0.281, *p* = 0.017), the left temporal pole of middle temporal gyrus (*r* = −0.270, *p* = 0.023), left inferior temporal gyrus (*r* = −0.254, *p* = 0.033), right inferior temporal gyrus (*r* = −0.243, *p* = 0.041), and right medial geniculate nucleus (*r* = −0.311, *p* = 0.008) in AD-OSA patients, respectively. AHI was not significantly correlated with the GMVs of other brain regions (Figure 6).

**Figure 7:**
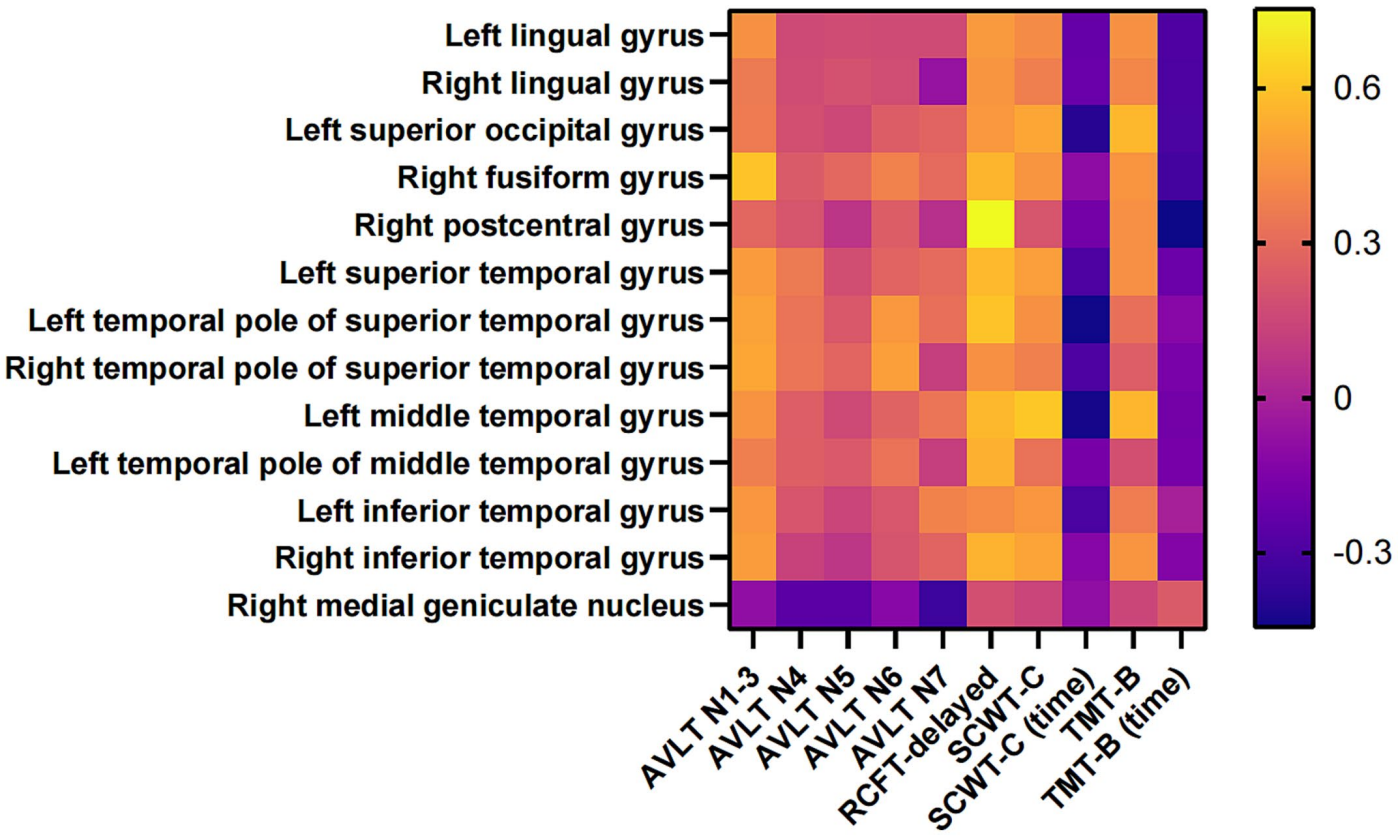
The correlation between cognitive function and the GMVs of ROIs in AD-OSA-MS group. AD-OSA-MS, Alzheimer’s disease with moderate and severe obstructive sleep apnea; GMV, gray matter volume; AVLT, Auditory Verbal Learning Test; RCFT, Rey–Osterrieth Complex Figure Test; SCWT, Stroop Color and Word Test; TMT, Trail Making Test.

Based on the findings that the memory and executive function were significantly declined, and the GMVs in several regions were significantly decreased in AD-OSA-MS group, we further analyzed the correlation between memory, executive function and the GMVs associated with AHI in AD-OSA-MS patients. The results showed that the decreased GMVs in several brain regions associated with the aggravation of OSA was significantly correlated with memory decline and executive dysfunction in AD-OSA-MS group (all *p* < 0.05) (Figure 7 and Supplementary Table 3).

#### 3.7.2 WMV

The WMVs of 170 corresponding brain regions were compared between AD-OSA-M and AD-OSA-MS groups. Compared with AD-OSA-M group, AD-OSA-MS group exhibited significantly reduced WMVs in 22 brain regions, including right superior frontal gyrus, left middle frontal gyrus, bilateral opercular part of inferior frontal gyrus, left triangular part of inferior frontal gyrus, right rolandic operculum, bilateral postcentral gyrus, right superior parietal gyrus, left inferior parietal gyrus, left supramarginal gyrus, left precuneus, right paracentral lobule, left superior temporal gyrus, bilateral temporal pole of superior temporal gyrus, left middle temporal gyrus, left temporal pole of middle temporal gyrus, bilateral inferior temporal gyrus, right lobule III of cerebellar hemisphere, and left lateral posterior thalamic (all *p* < 0.05). There were no significant differences in the WMVs of other brain regions between the two groups (Table 5).

**Table 5:** Comparisons of the WMVs of ROIs between AD-OSA-M and AD-OSA-MS groups.

WMV	AD-OSA-M group (*n* = 42)	AD-OSA-MS group (*n* = 30)	*p*
Right superior frontal gyrus (mm^3^, mean ± SD)	7.73 ± 1.22	7.16 ± 1.14	0.049^*^
Left middle frontal gyrus (mm^3^, mean ± SD)	6.52 ± 1.05	5.97 ± 1.11	0.038^*^
Left opercular part of inferior frontal gyrus (mm^3^, mean ± SD)	2.04 ± 0.38	1.86 ± 0.33	0.048^*^
Right opercular part of inferior frontal gyrus (mm^3^, mean ± SD)	1.77 ± 0.33	1.59 ± 0.32	0.024^*^
Left triangular part of inferior frontal gyrus (mm^3^, mean ± SD)	4.54 ± 0.81	4.10 ± 0.75	0.021^*^
Right rolandic operculum (mm^3^, mean ± SD)	1.53 ± 0.25	1.40 ± 0.22	0.025^*^
Left postcentral gyrus (mm^3^, mean ± SD)	6.82 ± 1.05	6.30 ± 0.93	0.035^*^
Right postcentral gyrus (mm^3^, mean ± SD)	5.75 ± 0.79	5.25 ± 0.90	0.014^*^
Right superior parietal gyrus (mm^3^, mean ± SD)	1.67 ± 0.31	1.47 ± 0.29	0.009^**^
Left inferior parietal gyrus (mm^3^, mean ± SD)	3.12 ± 0.50	2.85 ± 0.57	0.038^*^
Left supramarginal gyrus (mm^3^, mean ± SD)	2.58 ± 0.46	2.30 ± 0.40	0.009^**^
Left precuneus (mm^3^, mean ± SD)	5.08 ± 0.86	4.57 ± 0.87	0.016^*^
Right paracentral lobule (mm^3^, mean ± SD)	1.48 ± 0.30	1.32 ± 0.30	0.029^*^
Left superior temporal gyrus (mm^3^, mean ± SD)	4.45 ± 0.77	4.02 ± 0.74	0.023^*^
Left temporal pole of superior temporal gyrus (mm^3^, mean ± SD)	0.74 ± 0.15	0.65 ± 0.16	0.015^*^
Right temporal pole of superior temporal gyrus (mm^3^, mean ± SD)	1.25 ± 0.29	1.10 ± 0.24	0.020^*^
Left middle temporal gyrus (mm^3^, mean ± SD)	8.13 ± 1.46	7.33 ± 1.57	0.032^*^
Left temporal pole of middle temporal gyrus (mm^3^, mean ± SD)	0.88 ± 0.20	0.76 ± 0.22	0.020^*^
Left inferior temporal gyrus (mm^3^, mean ± SD)	4.77 ± 0.96	4.27 ± 0.99	0.039^*^
Right inferior temporal gyrus (mm^3^, mean ± SD)	3.81 ± 0.72	3.43 ± 0.70	0.033^*^
Right lobule III of cerebellar hemisphere (mm^3^, mean ± SD)	0.23 ± 0.05	0.21 ± 0.03	0.037^*^
Left lateral posterior thalamic (mm^3^, mean ± SD)	0.03 ± 0.02	0.04 ± 0.02	0.013^*^

Data were presented as means ± SD. ^*^p < 0.05 and ^**^p < 0.01. WMV, white matter volume; AD-OSA-M, Alzheimer’s disease with mild obstructive sleep apnea; AD-OSA-MS, Alzheimer’s disease with moderate and severe obstructive sleep apnea; SD, standard deviation.

**Figure 8:**
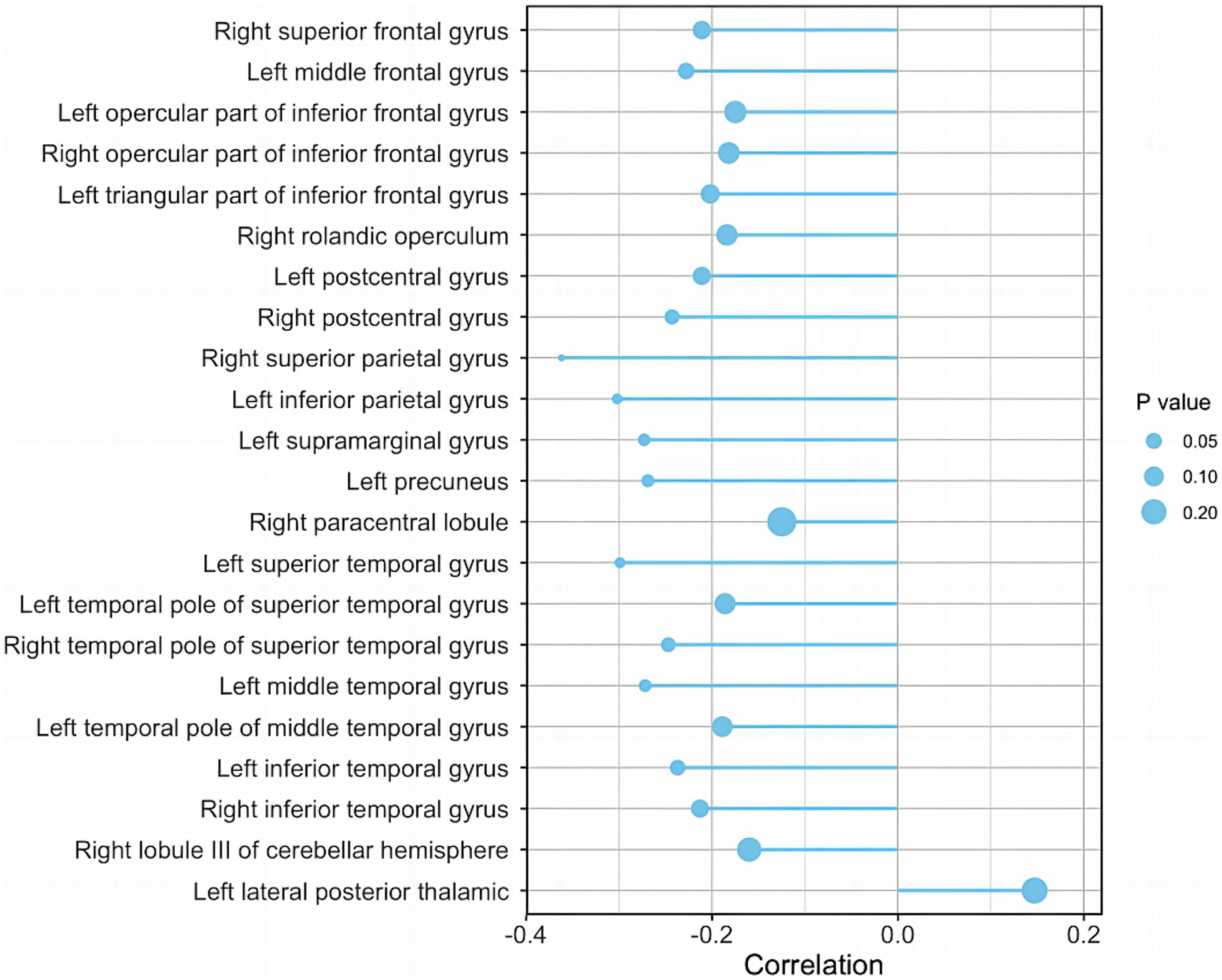
The correlation between AHI and the WMVs of ROIs in AD-OSA patients. AD-OSA, Alzheimer’s disease with obstructive sleep apnea; AHI, apnea–hypopnea index; WMV, white matter volume.

The correlations between AHI and WMV of the above 22 brain regions were analyzed in AD-OSA patients, respectively. It was presented that AHI level was significantly and negatively correlated with the WMVs of right postcentral gyrus (*r* = −0.243, *p* = 0.041), right superior parietal gyrus (*r* = −0.362, *p* = 0.002), left inferior parietal gyrus (*r* = −0.302, *p* = 0.010), left supramarginal gyrus (*r* = −0.273, *p* = 0.021), left precuneus (*r* = −0.269, *p* = 0.023), left superior temporal gyrus (*r* = −0.299, *p* = 0.011), the right temporal pole of superior temporal gyrus (*r* = −0.247, *p* = 0.038), left middle temporal gyrus (*r* = −0.272, *p* = 0.022), and left inferior temporal gyrus (*r* = −0.237, *p* = 0.047) in AD-OSA patients. AHI was not significantly correlated with the WMVs of other brain regions (Figure 8).

**Figure 9:**
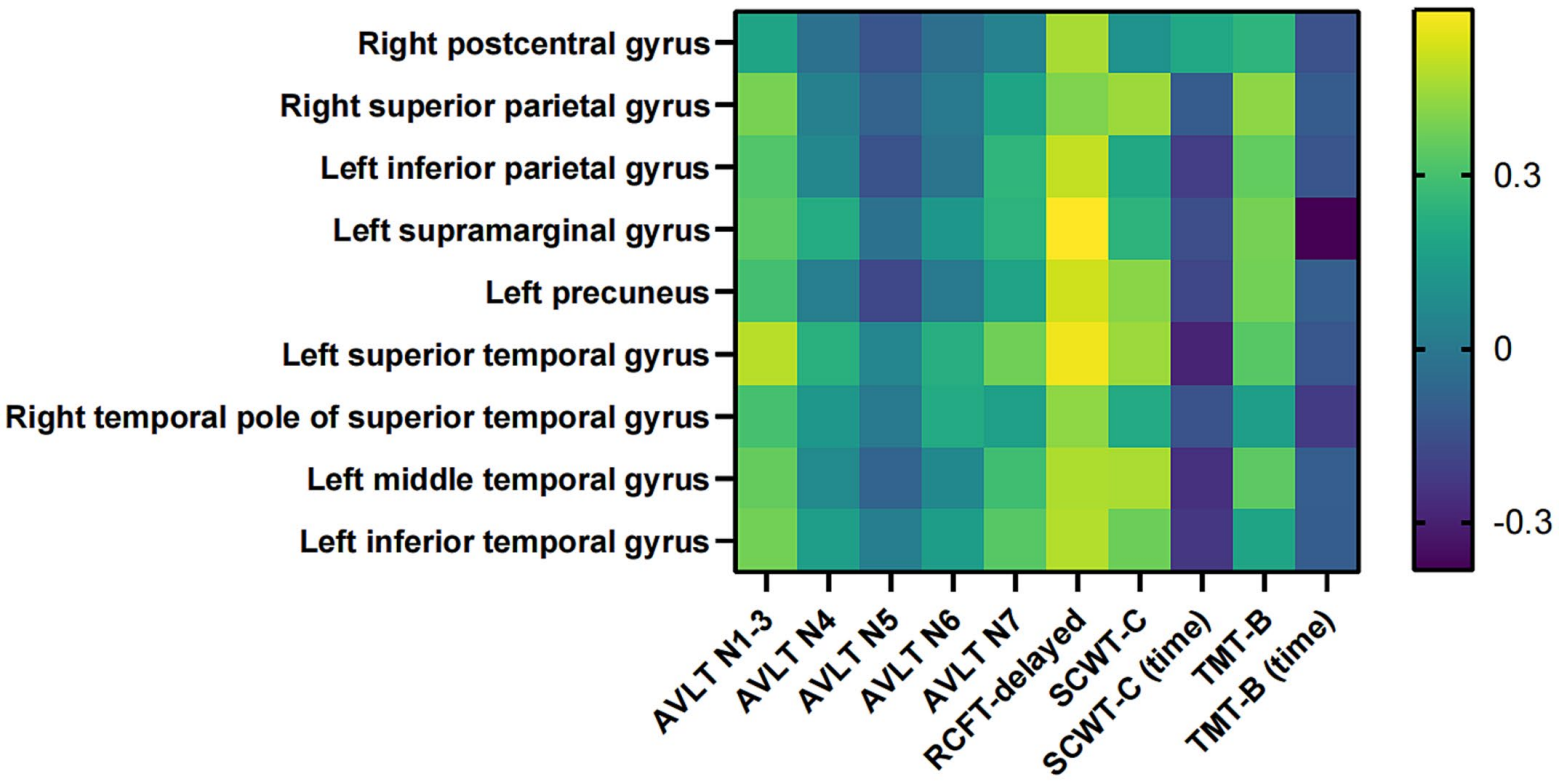
The correlation between cognitive function and the WMVs of ROIs in AD-OSA-MS group. AD-OSA-MS, Alzheimer’s disease with moderate and severe obstructive sleep apnea; WMV, white matter volume; AVLT, Auditory Verbal Learning Test; RCFT, Rey–Osterrieth Complex Figure Test; SCWT, Stroop Color and Word Test; TMT, Trail Making Test.

We further analyzed the correlation between memory, executive function and WMVs which associated by AHI in AD-OSA-MS patients. The results showed that in AD-OSA-MS patients, the decrease of WMVs in several brain regions with the aggravation of OSA was significantly correlated with the declined memory and executive function (all *p* < 0.05) (Figure 9 and Supplementary Table 4).

## 4 Discussion

### 4.1 The frequencies of different degrees of OSA in AD-OSA patients

A total of 94 AD-OSA patients were recruited in this study, and the frequency of AD-OSA-M and AD-OSA-MS were 52.13% and 47.87%, respectively, showing that AD-OSA-MS accounted for nearly half of AD-OSA (Table 1), which was similar to the previous studies presenting that AD-OSA-MS accounting for more than half of AD-OSA (Gaeta et al., 2020). A large 15-year follow-up study revealed that severe OSA was associated with the risk of AD, so it was of significance to intervene in severe OSA (Lutsey et al., 2018).

### 4.2 AD-OSA and demographic information

We found no difference in gender between AD-OSA-M and AD-OSA-MS groups. There were very few investigations on the demographic information of AD-OSA patients. One study compared demographic information between AD patients with mild to moderate OSA and severe OSA, showing a higher proportion of male patients in AD with severe OSA group (Gaeta et al., 2020). In AD patients with severe OSA, women have a higher proportion (Heinzer et al., 2015). Thus, gender difference in AD with severe OSA remains inconsistent, and is not significant in AD with moderate and severe OSA. Further large-scale epidemiological studies are warranted to validate these findings.

Previous studies on the relationship between OSA and BMI in cognitively normal individuals are common. However, there was only one observational study in AD-OSA patients, which reported that the severe OSA group had a higher BMI compared to mild to moderate OSA group (Gaeta et al., 2020). In our study, we categorized AD-OSA patients into AD-OSA-M and AD-OSA-MS groups. We found that AD-OSA-MS group had a significantly higher BMI than AD-OSA-M group (Table 1). This suggests that OSA may aggravate overweight in AD patients by potentially affecting lipid metabolism and raising cholesterol and blood lipid levels (Bonsignore, 2022). In turn, the increased BMI may also exacerbate OSA by contributing to the collapse of upper respiratory tract.

### 4.3 AD-OSA and cognitive function

A recent cross-sectional study found that AD patients with mild to moderate OSA did not show significant differences in the functions of overall cognition and individual cognitive domains compared with AD patients with severe OSA (Gaeta et al., 2020). In the current study, we divided AD-OSA patients into AD-OSA-MS and AD-OSA-M groups and found that AD-OSA-MS group exhibited seriously impaired memory and executive function compared with AD-OSA-M group (Table 2). After adjusting for confounding factors, the more severe OSA was still associated with worse memory and executive function (Figure 2). Variations in grouping and used rating scales may account for the differences in the impact of OSA on individual cognitive domains between our study and others. Therefore, we should pay more attention to the changes of cognitive domains in AD-OSA patients, particularly in AD-OSA-MS patients, and provide early intervention for patients to prevent further decline of cognitive function.

### 4.4 AD-OSA and AD biomarkers

There was only one study about Aβ42 level in CSF from AD patients with or without OSA, indicating no significant difference between the two groups over time (Bubu et al., 2019). However, it did not categorize OSA based on AHI. In the current study, we adopted AHI as the grouping criterion and found no significant difference in Aβ42 level in CSF between AD-OSA-M and AD-OSA-MS groups (Table 3). A previous study showed that chronic intermittent hypoxia, a characteristic of OSA, increased the activity of BACE1 and γ-secretase, leading to increased generation and deposition of Aβ (Zhang et al., 2019). However, in AD patients, Aβ deposition reaches to a plateau with time extending, thus, chronic intermittent hypoxia may play limited impact on further Aβ accumulation, which may explain why the severity of OSA exerts no significant impact on Aβ deposition.

P-tau is another key biomarker of AD and is closely related to the progression of disease. A previous study found no significant difference in P-tau level in CSF between AD patients with and without OSA over time, and did not analyze the relationship between OSA and different sites of P-tau (Bubu et al., 2019). In the current study, AD-OSA-MS group showed a prominently elevated P-tau 396 level in CSF compared with AD-OSA-M group (Table 3), not only that, after adjusting for confounding factors, the more severe OSA was still associated with higher P-tau 396 level (Figure 3). These findings suggest that the worsened OSA is correlated with the elevated P-tau 396 level in CSF, potentially contributing to the progression of AD. P-tau 396 may serve as a specific site of phosphorylation in AD-OSA-MS group, which specific mechanism requires further investigation in the future.

### 4.5 AD-OSA and CDK5

Until now, one study showed that CDK5 level in CSF from AD patients was decreased than compared with that from control subjects (Oláh et al., 2015), while two autopsy studies observed that CDK5 level was increased in the brains of AD patients compared with that of control (Lee et al., 1999; Sultana and Butterfield, 2007). Therefore, it is hypothesized that the decreased CDK5 level in CSF may be related to its deposition in brain. A previous study in mice found that hypoxia increased CDK5 expression in hippocampal neurons (Fang et al., 2019). There have been no studies on CDK5 in OSA patients. In the current study, we found for the first time that the CDK5 level in the CSF from AD-OSA-MS group was significantly decreased compared with that from AD-OSA-M group (Table 3). After adjusting for confounding factors, the more severe OSA was still associated with the decreased CDK5 level in the CSF (potential more deposition in brain) from AD-OSA patients (Figure 3). Thus, we speculate that the impact of chronic intermittent hypoxia on CDK5 in humans may be similar to that in mice, indicated by the increased CDK5 expression in brain.

### 4.6 AD-OSA and synaptic proteins

Synaptic proteins, including synaptophysin, synapsin I, and SNAP-25, have been studied in AD. Previous studies showed that the levels of these synaptic proteins were increased in the CSF from AD patients compared with healthy controls, but decreased in autopsy-derived AD brains. Therefore, we speculate that the elevated levels of synaptic proteins in CSF may be due to their release after synaptic injury (Reddy et al., 2005; Kivisäkk et al., 2022; Mirza and Zahid, 2018; Shimohama et al., 1997; McGrowder et al., 2021). However, there is a lack of studies on the changes of synaptic proteins in the CSF from AD-OSA patients. In the current study, we reported for the first time that synaptophysin was the only synaptic protein showing significant elevation in the CSF from AD-OSA-MS group compared with that from AD-OSA-M group (Table 3). After adjusting for confounding factors, the more severe OSA was still associated with elevated synaptophysin level in CSF (Figure 3). Previous studies showed that blood–brain barrier hyperpermeability and neuroinflammation caused damage to synaptic plasticity in OSA patients. Thus, chronic intermittent hypoxia caused by OSA might aggravate synaptic damage by intensifying neuroinflammation (McGrowder et al., 2021; Jelic and Le Jemtel, 2008; Kerner and Roose, 2016; Zhang et al., 2025). Mechanisms underlying OSA-induced synaptic damage need further investigation.

### 4.7 Relationship between CDK5 and P-tau 396 levels in CSF from AD-OSA patients

We further investigated the relationship between CDK5 and P-tau 396 levels in the CSF from AD-OSA patients. A previous study in metabolically active slices made from brains of adult rats reported that CDK5 expression in brain increased P-tau 396 level in brain (Sengupta et al., 2006). In the hippocampus of mice with systemic inflammation, CDK5 was activated and accompanied by increase P-tau 396 (Seo et al., 2017). Therefore, animal studies showed that OSA caused CDK5 activation via hypoxia and systemic inflammation and further increase P-tau 396 (Jelic and Le Jemtel, 2008). However, there was no related study in AD-OSA patients.

We speculate that the sites where CDK5 induces tau hyperphosphorylation may be associated with different triggers. It was showed that abnormal glycosylation-induced CDK5 promoted phosphorylating tau at Thr181, Ser199, Thr231, and Ser396 in the brains of AD patients (Weng et al., 2014). In the current study, we found a significant correlation between decreased CDK5 level and increased P-tau 396 level in the CSF from AD-OSA patients (Figure 4), indicating that OSA may activate CDK5 and was primarily associated with enhanced tau phosphorylation at the site of Ser396. Further investigation is needed to elucidate the specific mechanism.

### 4.8 Relationship between CDK5 and synaptophysin levels in CSF of AD-OSA patients

CDK5 is associated with synaptic plasticity (Cheung et al., 2006). In CDK5 mice, the increased Aβ level in brain mediated the loss of synaptophysin caused by CDK5 (Giusti-Rodríguez et al., 2011). In transgenic mice overexpressing human tau, inhibition of CDK5 expression increased synaptophysin level in brain (Seo et al., 2017), indicating that the increased tau level might also mediate the loss of synaptophysin caused by CDK5. In the current study, we found for the first time that the decreased CDK5 level was linked to the increased synaptophysin level in the CSF from AD-OSA patients (Figure 5). Therefore, we speculate that Aβ deposition or P-tau elevation may promote CDK5-mediated synaptic damage reflected mainly by synaptophysin loss in AD-OSA patients.

### 4.9 AD-OSA and GMV/WMV

#### 4.9.1 AD-OSA and GMV

Previous studies showed that the GMVs of bilateral anterior cingulate gyrus, hippocampus/parahippocampal gyrus, orbitofrontal cortex, left cerebellum, and frontotemporal region were decreased in OSA patients compared with healthy controls, but the severity of OSA was not graded (Weng et al., 2014; Huang et al., 2019). In the current study, AD-OSA-MS group had more extensive GMV reduction in 17 brain regions than AD-OSA-M group (Table 4). Among these brain regions, 13 regions showed significant correlations with the severity of OSA, including parts of occipital lobe, temporal lobe, sensory cortex, and thalamus (Figure 6). Chronic intermittent hypoxia in AD-OSA patients may result in neuronal degeneration and death, leading to gray matter atrophy. With the aggravation of OSA, GMV atrophy was related to memory decline and executive dysfunction (Figure 7). Larger clinical trials are needed in the future to provide further validation.

#### 4.9.2 AD-OSA and WMV

A previous study showed no significant difference in WMV between OSA patients and healthy controls, but the sample size was small (Huynh et al., 2014). In the current study, we found for the first time that AD-OSA-MS group had extensive WMV reduction in 22 brain regions compared with AD-OSA-M group (Table 5), of which, 9 brain regions, mainly including parts of sensory cortex, parietal lobe, and temporal lobe, were significantly correlated with the severity of OSA (Figure 8). The decreased WMVs might be related to myelin injury caused by decreased cerebral perfusion during hypoxia and apnea (Kumar et al., 2014). With the aggravation of OSA, WMV atrophy was also related to memory declined and executive dysfunction (Figure 9). Interestingly, the WMV of lateral posterior thalamic nucleus in AD-OSA-MS group was larger than that in AD-OSA-M group, but it was not correlated with AHI, which might be related to the increase of compensatory myelination after chronic intermittent hypoxia. Furthermore, we found that the impact of OSA on GMV was more pronounced than that on WMV in AD-OSA patients. We speculate that OSA may has a wider effect on neurons than on nerve fibers in AD-OSA patients. Larger sample sizes are needed for further validation in the future.

### 4.10 Limitations

This study had the following limitations. Firstly, it was very difficult to obtain CSF samples from the elderly population, and the number of patients who completed both CSF test and imaging examination was relatively insufficient. Secondly, this cross-sectional study was not enough to explain the causal relationship between OSA and the variables measured, and a prospective investigation is needed in the future. Thirdly, as the compliance to PSG of AD patients was relatively low, OSA was diagnosed with PSG and portable overnight actigraphy in this study. In the future, we will further investigate the impact of different sleep monitoring devices on the outcomes of patients with OSA and establish an efficient and practical sleep monitoring approach suitable for AD patients.

## 5 Conclusion

AD-OSA patients have a high frequency of AD-OSA-MS. OSA exacerbates overweight and accelerates memory decline and executive dysfunction with increased severity in AD-OSA patients. OSA exacerbation is associated with P-tau 396 elevation and CDK5 decline in CSF (potential upregulation in brain), synaptic disruption, and brain atrophy in AD-OSA patients. CDK5 decline in CSF is associated with P-tau 396 elevation and synaptic loss in AD-OSA patients. In clinical practice, OSA and its severity should be routinely monitored and timely intervened to prevent AD progression, and CDK5 may represent a potential therapeutic target for the individuals with comorbid AD and OSA.

## Data Availability

The raw data supporting the conclusions of this article will be made available by the authors, without undue reservation.

## Footnotes

^1^ https://www.xiantao.love/
