# Supplementary material for: An investigation on Alzheimer’s disease with obstructive sleep apnea: alterations of cognitive function, roles of cyclin-dependent kinase 5 and changes of brain structure
Source: Front Aging Neurosci. 2025 Dec 1;17:1552535. doi: 10.3389/fnagi.2025.1552535 (PMC12702912; doi:10.3389/fnagi.2025.1552535)
Supplement: Supplementary file 1 [file Table_1.docx]

**Supplementary Table 1 The association between AHI and cognitive function in AD-OSA patients**

|  | **Unadjusted** | | **Adjusted** | |
| --- | --- | --- | --- | --- |
|  | **β (95% CI)** | ***P*** | **β (95% CI)** | ***P*** |
| AVLT-N7 (points) | -0.020 (-0.037, -0.003) | 0.019* | -0.020 (-0.039, -0.002) | 0.034* |
| SCWT-C (points) | -0.005 (-0.023, 0.013) | 0.597 | -0.007 (-0.028, 0.014) | 0.516 |
| SCWT-C (time) (seconds) | 0.024 (0.007, 0.042) | 0.007** | 0.027 (0.007, 0.047) | 0.008** |

Notes: age, gender, and BMI were adjusted. **P* < 0.05; ***P* < 0.01. Abbreviation: AD-OSA, Alzheimer’s disease with obstructive sleep apnea; AHI, apnea–hypopnea index; AVLT, Auditory Verbal Learning Test; SCWT, Stroop Color and Word Test; β, beta; CI, confidence interval.

**Supplementary Table 2 The association between AHI and the levels of P-tau 396, CDK5, and synaptophysin in the CSF from AD-OSA patients**

|  | **Unadjusted** | | **Adjusted** | |
| --- | --- | --- | --- | --- |
|  | **β (95% CI)** | ***P*** | **β (95%CI)** | ***P*** |
| P-tau 396 (pg/ml) | 0.016 (0.000, 0.032) | 0.054 | 0.020 (0.003, 0.038) | 0.024* |
| CDK5 (ng/ml) | -0.018 (-0.036, 0.001) | 0.058 | -0.028 (-0.047, -0.009) | 0.004** |
| synaptophysin (pg/ml) | 0.036 (0.019, 0.053) | <0.001** | 0.036 (0.018, 0.053) | <0.001** |

Notes: age, gender, and BMI were adjusted. **P* < 0.05; ***P* < 0.01. Abbreviation: AD-OSA, Alzheimer’s disease with obstructive sleep apnea; AHI, apnea–hypopnea index; P-tau, phosphorylated tau; CDK5, cyclin-dependent kinase 5; β, beta; CI, confidence interval.

**Supplementary Table 3 The correlation between cognitive function and GMVs of ROIs in AD-OSA-MS group**

|  |  | AVLT N1-3 | AVLT N4 | AVLT N5 | AVLT N6 | AVLT N7 | RCFT-  delayed | SCWT-C | SCWT-C (time) | TMT-B | TMT-B (time) |
| --- | --- | --- | --- | --- | --- | --- | --- | --- | --- | --- | --- |
| Left lingual gyrus | r | 0.441 | 0.164 | 0.174 | 0.168 | 0.169 | 0.470 | 0.420 | -0.222 | 0.439 | -0.285 |
|  | *P* | 0.017* | 0.395 | 0.367 | 0.385 | 0.380 | 0.013* | 0.029* | 0.308 | 0.017* | 0.135 |
| Right lingual gyrus | r | 0.358 | 0.175 | 0.195 | 0.176 | -0.068 | 0.457 | 0.375 | -0.207 | 0.404 | -0.294 |
|  | *P* | 0.056 | 0.365 | 0.311 | 0.361 | 0.726 | 0.017* | 0.054 | 0.344 | 0.030* | 0.121 |
| Left superior occipital gyrus | r | 0.360 | 0.189 | 0.157 | 0.244 | 0.273 | 0.463 | 0.512 | -0.394 | 0.564 | -0.299 |
|  | *P* | 0.055 | 0.327 | 0.417 | 0.203 | 0.151 | 0.015* | 0.006** | 0.063 | 0.001** | 0.116 |
| Right fusiform gyrus | r | 0.602 | 0.236 | 0.288 | 0.384 | 0.297 | 0.560 | 0.456 | -0.096 | 0.449 | -0.317 |
|  | *P* | < 0.001** | 0.218 | 0.130 | 0.040* | 0.117 | 0.002** | 0.017* | 0.662 | 0.015* | 0.094 |
| Right postcentral gyrus | r | 0.283 | 0.211 | 0.078 | 0.246 | 0.056 | 0.753 | 0.209 | -0.181 | 0.436 | -0.444 |
|  | *P* | 0.137 | 0.272 | 0.689 | 0.199 | 0.774 | < 0.001** | 0.294 | 0.408 | 0.018* | 0.016* |
| Left superior temporal gyrus | r | 0.474 | 0.356 | 0.184 | 0.270 | 0.300 | 0.573 | 0.484 | -0.295 | 0.435 | -0.203 |
|  | *P* | 0.009** | 0.058 | 0.339 | 0.156 | 0.114 | 0.002** | 0.011* | 0.172 | 0.019* | 0.290 |
| Left temporal pole of superior temporal gyrus | r | 0.498 | 0.335 | 0.223 | 0.465 | 0.321 | 0.602 | 0.443 | -0.442 | 0.320 | -0.117 |
|  | *P* | 0.006** | 0.076 | 0.244 | 0.011* | 0.090 | 0.001** | 0.021* | 0.035* | 0.091 | 0.544 |
| Right temporal pole of superior temporal gyrus | r | 0.510 | 0.339 | 0.276 | 0.490 | 0.121 | 0.441 | 0.380 | -0.292 | 0.250 | -0.159 |
|  | *P* | 0.005** | 0.072 | 0.148 | 0.007** | 0.532 | 0.021* | 0.051 | 0.176 | 0.191 | 0.411 |
| Left middle temporal gyrus | r | 0.446 | 0.249 | 0.162 | 0.269 | 0.338 | 0.566 | 0.613 | -0.433 | 0.562 | -0.180 |
|  | *P* | 0.015* | 0.193 | 0.400 | 0.158 | 0.073 | 0.002** | < 0.001** | 0.039* | 0.002** | 0.350 |
| Left temporal pole of middle temporal gyrus | r | 0.374 | 0.251 | 0.230 | 0.332 | 0.121 | 0.542 | 0.327 | -0.166 | 0.187 | -0.168 |
|  | *P* | 0.046* | 0.189 | 0.229 | 0.078 | 0.532 | 0.004** | 0.096 | 0.450 | 0.330 | 0.382 |
| Left inferior temporal gyrus | r | 0.458 | 0.216 | 0.147 | 0.219 | 0.390 | 0.419 | 0.458 | -0.303 | 0.365 | -0.010 |
|  | *P* | 0.012* | 0.261 | 0.447 | 0.255 | 0.036* | 0.030* | 0.016* | 0.160 | 0.051 | 0.957 |
| Right inferior temporal gyrus | r | 0.478 | 0.131 | 0.085 | 0.207 | 0.270 | 0.547 | 0.506 | -0.121 | 0.451 | -0.131 |
|  | *P* | 0.009** | 0.500 | 0.662 | 0.281 | 0.157 | 0.003** | 0.007** | 0.582 | 0.014* | 0.499 |
| Right medial geniculate nucleus | r | -0.087 | -0.252 | -0.256 | -0.111 | -0.340 | 0.188 | 0.144 | -0.085 | 0.152 | 0.236 |
|  | *P* | 0.654 | 0.187 | 0.180 | 0.565 | 0.071 | 0.347 | 0.473 | 0.700 | 0.431 | 0.217 |

Notes: **P*<0.05; ***P*<0.01. Abbreviation: AD-OSA-MS, Alzheimer’s disease with moderate and severe obstructive sleep apnea; GMV, grey matter volume; AVLT, Auditory Verbal Learning Test; RCFT, Rey-Osterrieth Complex Figure Test; SCWT, Stroop Color and Word Test; TMT, Trail Making Test.

**Supplementary Table 4 The correlation between cognitive function and WMVs of ROIs in AD-OSA-MS group**

|  |  | AVLT N1-3 | AVLT N4 | AVLT N5 | AVLT N6 | AVLT N7 | RCFT-  delayed | SCWT-C | SCWT-C (time) | TMT-B | TMT-B (time) |
| --- | --- | --- | --- | --- | --- | --- | --- | --- | --- | --- | --- |
| Right postcentral gyrus | r | 0.180 | -0.026 | -0.130 | -0.033 | 0.044 | 0.460 | 0.109 | 0.193 | 0.247 | -0.141 |
|  | *P* | 0.350 | 0.893 | 0.501 | 0.865 | 0.820 | 0.016* | 0.589 | 0.377 | 0.196 | 0.465 |
| Right superior parietal gyrus | r | 0.389 | 0.037 | -0.081 | 0.007 | 0.181 | 0.400 | 0.445 | -0.108 | 0.421 | -0.105 |
|  | *P* | 0.037* | 0.849 | 0.677 | 0.971 | 0.347 | 0.039* | 0.020* | 0.625 | 0.023* | 0.587 |
| Left inferior parietal gyrus | r | 0.324 | 0.065 | -0.138 | -0.012 | 0.249 | 0.499 | 0.196 | -0.210 | 0.353 | -0.128 |
|  | *P* | 0.086 | 0.737 | 0.476 | 0.951 | 0.193 | 0.008** | 0.328 | 0.336 | 0.060 | 0.509 |
| Left supramarginal gyrus | r | 0.338 | 0.207 | -0.028 | 0.125 | 0.238 | 0.586 | 0.242 | -0.158 | 0.384 | -0.382 |
|  | *P* | 0.073 | 0.281 | 0.884 | 0.519 | 0.213 | 0.001** | 0.224 | 0.471 | 0.040* | 0.041* |
| Left precuneus | r | 0.297 | 0.029 | -0.176 | 0.008 | 0.179 | 0.516 | 0.415 | -0.183 | 0.379 | -0.101 |
|  | *P* | 0.118 | 0.879 | 0.362 | 0.967 | 0.352 | 0.006** | 0.031* | 0.404 | 0.043* | 0.602 |
| Left superior temporal gyrus | r | 0.482 | 0.228 | 0.058 | 0.223 | 0.377 | 0.567 | 0.443 | -0.290 | 0.332 | -0.126 |
|  | *P* | 0.008** | 0.234 | 0.765 | 0.245 | 0.044* | 0.002** | 0.021* | 0.180 | 0.079 | 0.515 |
| Right temporal pole of superior temporal gyrus | r | 0.301 | 0.129 | 0.007 | 0.205 | 0.164 | 0.424 | 0.204 | -0.138 | 0.154 | -0.217 |
|  | *P* | 0.112 | 0.506 | 0.970 | 0.286 | 0.395 | 0.027* | 0.308 | 0.529 | 0.425 | 0.259 |
| Left middle temporal gyrus | r | 0.358 | 0.076 | -0.075 | 0.069 | 0.287 | 0.469 | 0.463 | -0.252 | 0.342 | -0.100 |
|  | *P* | 0.057 | 0.696 | 0.699 | 0.722 | 0.131 | 0.014* | 0.015* | 0.246 | 0.069 | 0.604 |
| Left inferior temporal gyrus | r | 0.380 | 0.154 | 0.026 | 0.153 | 0.332 | 0.477 | 0.371 | -0.227 | 0.180 | -0.102 |
|  | *P* | 0.042* | 0.426 | 0.892 | 0.428 | 0.079 | 0.012* | 0.057 | 0.298 | 0.351 | 0.598 |

Notes: **P*<0.05; ***P*<0.01. Abbreviation: AD-OSA-MS, Alzheimer’s disease with moderate and severe obstructive sleep apnea; WMV, white matter volume; AVLT, Auditory Verbal Learning Test; RCFT, Rey-Osterrieth Complex Figure Test; SCWT, Stroop Color and Word Test; TMT, Trail Making Test.
